# Why can pulmonary vein stenoses created by radiofrequency catheter ablation worsen during and after follow-up ? A potential explanation

**DOI:** 10.1186/1749-8090-3-24

**Published:** 2008-05-05

**Authors:** Pierre-André Doriot, Pierre-André Dorsaz, Dipen Chandrakant Shah

**Affiliations:** 1 Cardiology Department, University Hospital of Geneva, Geneva, Switzerland

## Abstract

**Background:**

Radiofrequency catheter ablation of excitation foci inside pulmonary veins (PV) generates stenoses that can become quite severe during or after the follow-up period. Since severe PV stenoses have most often disastrous consequences, it would be important to know the underlying mechanism of this temporal evolution. The present study proposes a potential explanation based on mechanical considerations.

**Methods:**

we have used a mathematical-physical model to examine the cyclic increase in axial wall stress induced in the proximal (= upstream), non-stenosed segment of a stenosed pulmonary vein during the forward flow phases. In a representative example, the value of this increase at peak flow was calculated for diameter stenoses (DS) ranging from 1 to 99%.

**Results:**

The increase becomes appreciable at a DS of roughly 30% and rise then strongly with further increasing DS value. At high DS values (e.g. > 90%) the increase is approximately twice the value of the axial stress present in the PV during the zero-flow phase.

**Conclusion:**

Since abnormal wall stresses are known to induce damages and abnormal biological processes (e.g., endothelium tears, elastic membrane fragmentations, matrix secretion, myofibroblast generation, etc) in the vessel wall, it seems plausible that the supplementary axial stress experienced cyclically by the stenotic and the proximal segments of the PV is responsible for the often observed progressive reduction of the vessel lumen after healing of the ablation injury. In the light of this model, the only potentially effective therapy in these cases would be to reduce the DS as strongly as possible. This implies most probably stenting or surgery.

## Introduction

**Figure 1:**
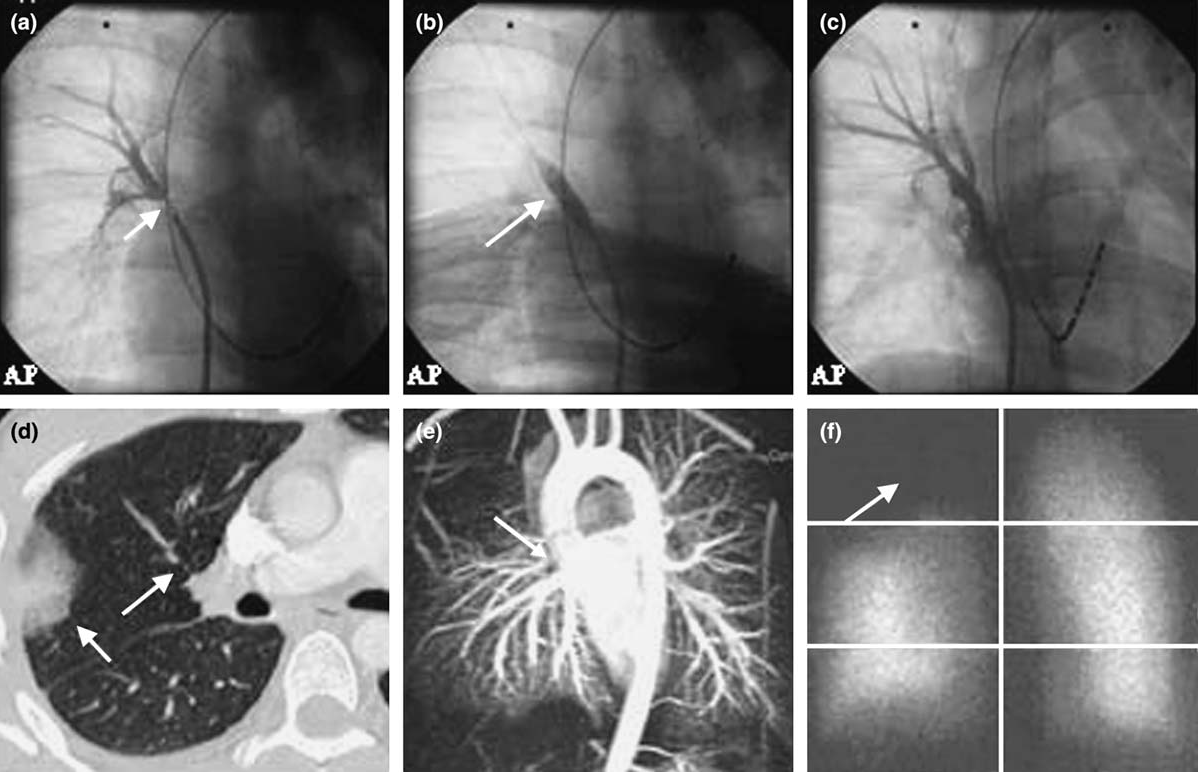
Images of a right superior pulmonary vein stenosis (Reproduced with permission from ref. [14]): (a) Angiographic view of the high grade stenosis (arrow). (b) Balloon dilatation (arrow). (c) Result after dilatation and stenting. (d) Pre-interventional computed tomographic scanning of the stenosis (arrow), including infiltration in the dependent lung lobe (arrow). (e) Three-dimensional magnetic resonance reconstruction of the stenosis (arrow). (f) Lung scan showing perfusion deficit in the right upper field (arrow).

Radiofrequency (RF) catheter ablation of excitation foci in extraparenchymal pulmonary veins (PV), or electrical isolation of these veins from the left atrium, is increasingly applied to suppress atrial fibrillation (AF). However, several studies have shown that these techniques sometimes create severe stenoses or even total occlusions in the concerned veins [1-14]. Fig. 1 shows such a stenosis; further illustrations can be found for instance in ref. [2] or on the Web. The stenosis rates reported in the literature vary from 0 percents to more than 40% [6-9,15-18]. These discrepancies are considerable but have actually simple causes: a) The definition of "stenosis" is not the same in all studies (e.g. > 25, 30, 50, or even 70% diameter reduction) [16-18]. b) The stenosis rate is sometimes defined per patient instead of per treated PV [6,17]. c) The severity of the created stenoses depends on many parameters, such as number of ablated foci, extent of the treated intraluminal zone, number and duration of the ablation pulses, temperature and temperature distribution generated by the RF-field in the vessel wall, etc. d) Stenoses without clinical repercussions remain undiscovered if specific examinations (CT, MRI, or echocardiography) are not performed. e) Many stenoses are misdiagnosed even when the clinical symptoms are evident [10,17,19]. f) Stenoses are underreported [19]. Arentz et al. for instance found that the rate of PV stenosis (defined as > 50% diameter reduction) is considerably higher than the rate suggested by clinical assessments [7]. In their study, only 3 patients out of 47 had symptoms suggestive of PV stenosis, whereas systematic surveillance by transoesophageal Doppler-echo and/or magnetic resonance imaging revealed PV stenosis or complete occlusion in 13 patients (28%).

The presence of an important PV stenosis at follow-up is always a serious complication because of the progressive repercussions these stenoses can have on pulmonary circulation. It is indeed well known in corrective cardiovascular surgery that the evolution of patients presenting congenital PV stenoses is practically never favorable, due (mainly) to the progression of stenosis severity with the years [20,21]. This probably also applies to some stenoses created by RF ablation. Several authors have indeed reported a progression of PV stenoses during follow-up [4,6-9,12-14]. For instance, Taylor et al. measured PV stenoses angiographically in dogs at intervals of 2 to 4 weeks, 6 to 8 weeks, or 10 to 14 weeks, and observed an increase of stenosis severity with time [4]. Similarly, Arentz et al. performed angiographic and MRI measurements in humans 2 years after a RF ablation procedure and came to the same conclusion [7]. Some moderate stenoses may, however, also be smaller at follow-up than shortly after the procedure. This is of course not surprising because the inflammatory processes induced by the thermal injury in the vessel wall [8] will ultimately vanish. Nevertheless, it is obvious that the creation of PV stenoses by the radio frequency ablation process is an important issue, even if the true rate is not exactly known, and even if this latter were of only a few percents.

Today, the risk of creating a severe PV stenosis is still non negligible despite the methodological progresses accomplished in the late four years. It is therefore important to find out why PV stenoses usually do not regress, and why many will even progress. The phenomenon responsible for the apparition of a stenosis during or shortly after the intervention has been elucidated and does not seem to play a role in the late evolution of PV stenoses [10,15,22]. The late evolution has also been studied, but only from a phenomenological point of view [4]. We could not find any potential explanation of the cause of this evolution, despite an extensive literature search.

A well-known effect a severe stenosis has on the affected vessel is, of course, the cyclic (and sometimes also chronic) pressure rise that is generated in the vascular net upstream of the stenosis. This pressure increase induces media and intima thickening (among else), and may ultimately lead to pulmonary insufficiency. Wall thickening can be attributed to the changes in circumferential wall stress induced by the cyclic or chronic increase of the intravascular pressure but these stress changes cannot well explain why stenoses do not regress or even progress. Moreover, PV stenoses are not likely to induce high circumferential stresses in the upstream vessels because the increase in capacitance of the pulmonary venous net during systole (which is roughly equal to the stroke volume) is due to a deformation of the lumens from a slit form into a circular one, and not to a circumferential elongation of the vein walls [23]. In contrast thereto, a cyclic increase of axial wall stress in the vein segment situated upstream of the stenosis, generated by this stenosis during the (forward) flow phases, might play a still unsuspected role. In this contribution, we propose a potential explanation for the progression of PV stenoses that based on this effect of stenoses on axial wall stress. Thereby, the main aspect is not the mathematical-physical model we used but the conclusions it leads to.

## Methods

### The model

**Figure 2:**
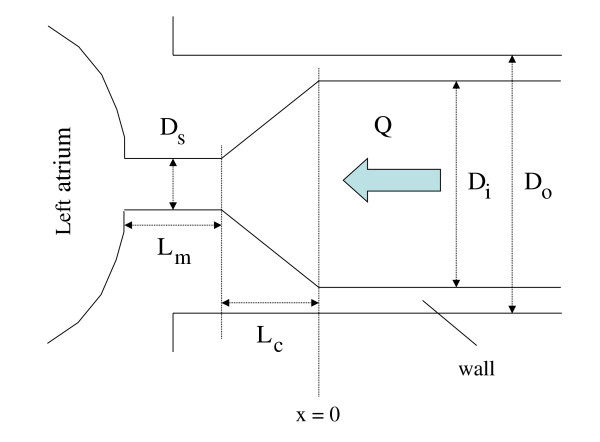
Schematic illustration of the considered pulmonary vein stenosis. Since the dominant parameter from a hemodynamical-mechanical point of view is the lumen reduction, this drawing is also valid for stenoses with a different morphology.

We consider a stenosis on a PV. For simplicity, this stenosis is assumed to be at the vein ostium (Fig. 2). The basic idea is that stenoses pull at the proximal (undiseased) vessel segment during the (forward) venous flow phases, increasing thus the axial wall stress in this vessel segment cyclically beyond its "normal" value. In the following, this "normal" stress is considered to be constant throughout the cardiac cycle, and to have roughly the value it had in the undiseased vein. Thus, the supplementary axial stress generated cyclically by the stenosis adds to the "normal" stress. "Peak flow" will denote peak venous flow through the corresponding lung lobe.

We want to estimate the increase in axial wall stress at the stenosis entrance (cross section x = 0 in Fig. 2) at peak flow, and to compare this increase to "normal" axial stress. Thereto, we have to calculate the pressure drop (ΔP) across the stenosis at peak flow, and the resulting axial force F in the wall cross section x = 0. Division of this force F by the cross sectional area of the wall yields then the supplementary axial stress generated by the stenosis. We consider the situation at peak flow because F is approximately maximal at that time, due to the fact that the pressure drop across the stenosis is maximal at that time.

The pressure drop ΔP can be calculated using formulae proposed by Back et al. [24] (See Appendix). One has to know thereto the inner vessel diameter (D_i_, see Fig. 2), the minimum inner diameter (D_s_) (or, alternatively, the degree of stenosis DS), the respective lengths of constriction cone (L_c_) and stenosis throat (L_m_), the blood density (ρ), the blood viscosity (η), and the peak flow (Q).

Since flow Q depends on the pressure available to drive the blood through the considered lung lobe and the stenosis, on the hemodynamical resistance R of this lobe at peak (venous) flow, and on the resistance opposed by the stenosis, the value of Q cannot be chosen freely, except for DS = 0%. We make therefore use of the facts that the pattern of flow in the large extraparenchymal PV is pulsatile, and that the pulsatility is dominated by the changes in left atrial pressure that take place throughout the cardiac cycle. These facts were demonstrated by Rajagopalan et al. [23] who also pointed out that all venous flow curves recorded in their experiments resembled inverted left atrial pressure curves. Based on these observations, we can approximate the relationship between pressures and peak flow in the considered lung lobe by the two equations:

P_PA _- P_LA _= Q_o _R_o _and P_PA_- P_LA _= Q R + ΔP(Q).

P_PA _is the value of pulmonary arterial pressure at the time of minimal left atrial pressure P_LA_. Based on the several pressure curves displayed in publications of the same authors [23,25,26], we take the pressure P_PA _equal to the mean pulmonary artery pressure. The difference P_PA _- P_LA _is thus the driving pressure at the time of peak flow. Q_o _is the peak flow one would have at that time in absence of the stenosis (DS = 0%), and R_o _is the hemodynamical resistance of the considered lung lobe for this flow (and at that time). Q is the actual peak flow when the stenosis is present (DS > 0%); it depends, of course, on the corresponding hemodynamical resistance R of the considered lobe. According to Rajagopalan et al. [25,26], all involved pulmonary arteries, arterioles, capillaries, venules, and veins have a more or less circular cross section at peak flow. At that time, pulmonary resistance is therefore minimal. Since pulmonary veins do not have appreciable vasoconstriction capabilities, and since they are also relatively inextensible [27], we can assume for simplicity that R has the same value for all DS that will be considered (1% to 99% diameter reduction). We set thus R = R_o _for all DS values. The last term of the second equation, the pressure drop ΔP(Q), is a function of flow Q, as already mentioned.

These equations, together with the formulas of Back et al. [24], lead to a quadratic equation that allows to calculate flow Q for any given DS value (Equation 8 in the Appendix). The freely choosable parameters are: the pressures P_PA _and P_LA_, peak flow Q_o_, the blood density and viscosity, and the stenosis geometry. The corresponding value of force F is then calculated using the corresponding pressure drop ΔP(Q), the pressure P_LA_, and the formulae of Back et al. The calculation is rather complex and is described in detail in [28].

Since there is no evidence that stenoses induce a permanent axial elongation of the affected PV, there is no reason to assume that axial stress in the wall cross section x = 0 decreases below the "normal" value during flow diastole. Consequently, one can consider that axial wall stress is simply the sum of "normal" axial stress and supplementary axial stress, as assumed above.

Finally, we can also calculate the ratio "increased axial wall stress over normal axial wall stress" at the entrance of the stenosis. Contrary to absolute stress values, this ratio does not depend on the actual area value of the wall cross section.

### Numerical example

In this section, we consider for illustration a representative PV, and we use the model described in the preceding section to calculate the cyclic increase in axial wall stress that can be expected at the entrance of the stenosis for DS values ranging from 1% and 99%.

Based on the literature we chose for the parameters needed for the calculation of the pressure drop ΔP a normal inner diameter (D_i_) of 10 mm at the stenosis entrance [7,8,14,27,29] and a maximal flow velocity (over the lumen) of 60 cm/s at peak flow Q_o _(DS = 0%) [5,14,15,18,30,31]. Assuming that the velocity profile at peak flow is parabolic in absence of a stenosis, the value of Q_o _is then 23.6 ml/s.

As already explained in the preceding section, we also assume that the resistance R (= R_o_) of the considered lung lobe is independent of the DS, and that it is equal, at peak flow, to the product "mean pulmonary artery pressure (P_PA_) minus minimal left atrial pressure (P_LA_)" times "flow Q_o_". With P_PA _= 20 mmHg and P_LA _= 0 mmHg (see [[32], p. 1700]), we have thus R = (P_PA _- P_LA_)/Q_o _= 20 mmHg/23.6 ml/s. Introduction of this value into equation 8 allows to calculate then peak flow Q through the considered lung lobe in dependence of the DS value (1% to 99%).

For the calculation of the axial wall force F associated to a particular DS value, we use the value of peak flow Q calculated for this DS value, and we determine the corresponding pressure drop ΔP(Q) across the stenosis. We add then ΔP(Q) to the minimal left atrial pressure P_LA _(0 mmHg) to obtain the pressure at the stenosis entrance at peak flow Q. The value of F is then obtained as described in [28].

In order to calculate the increase in axial wall stress generated by the force F, we have to chose a value for the relative wall thickness σ of the vein at the stenosis entrance (σ = D_o_/D_i_, where D_o _and D_i _are the outer and inner diameters of the intact vein segment, see Fig. 2). Based on various publications, we choose an absolute wall thickness of 0.2 mm [26,33,34]. Together with D_i _= 10 mm, this yields then a relative wall thickness of 1.04. Division of the F values obtained for the particular DS values (1% to 99%) yields then the values of the supplementary axial wall stress in function of the actual DS.

To compare the increase in axial stress to the "normal" axial stress that was present in the vein wall before the apparition of the stenosis (and which is still the axial wall stress of the non stenotic vein segment during the zero flow phase), we need this "normal" stress value. Since values for PV are not available in the literature, we make the (reasonable) assumption that "normal" axial stress has the value one would obtain by inflating in vitro an excised, occluded segment of the vein with a pressure equal to the mean in vivo intravascular pressure. In the present example, this pressure is practically equal to the mean pressure of the left atrium (P_LA, mean_). With a value P_LA, mean _of 8 mmHg [[32], p. 114], "normal" axial wall stress is then 13 kPa.

## Results

**Figure 3:**
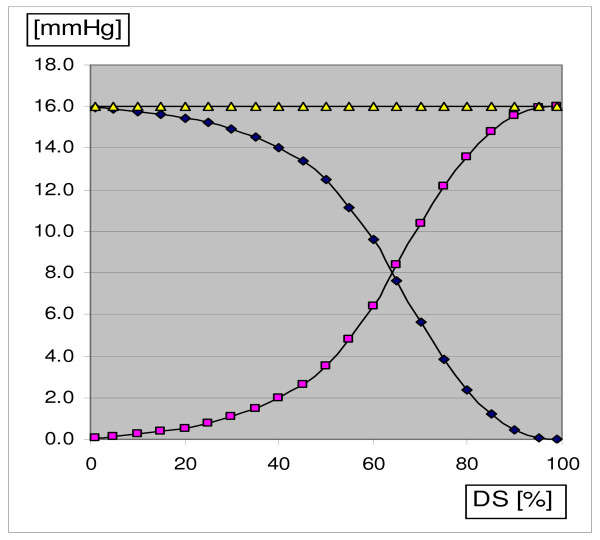
Pressure drops in the considered lung lobe (curve falling from the left to the right) and across the stenosis (curve rising from the left to the right) in function of increasing degree of diameter stenosis (DS). The sum of the two pressure drops (horizontal line) is constant, as imposed by the model.

Fig. 3 shows the pressure falls in the considered lung lobe and across the stenosis as functions of the parameter DS. The sum of the two pressure drops is constant, as imposed by the model. At high DS values, the pressure in the proximal (= upstream) segment adjacent to the stenosis is much greater than at low DS values. With regard to circumferential stress, this is probably not dramatic since the pressure in this segment climbs anyway to about 13 mmHg during atrial systole after suppression of the atrial fibrillation (Systolic left atrial pressure ranges from 6 to 20 mmHg, see [[32], p. 114]).

**Figure 4:**
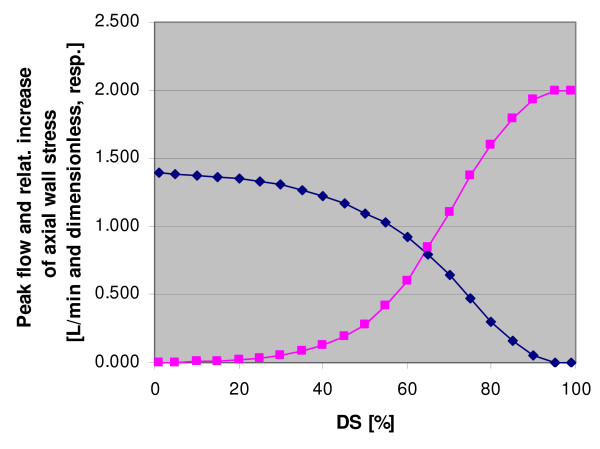
Value of peak flow Q in l/min (curve falling from the left to the right) and ratio "axial stress at peak flow at the stenosis entrance" over "normal axial stress" (curve rising from the left to the right) for diameter stenoses ranging from 1% to 99%. At DS = 50%, the flow reduction is already 25%. At DS = 100%, flow Q is, of course, zero. The stress increase begins at roughly 30% diameter reduction and reaches, in this example, twice the value of "normal" stress at high DS values.

Fig. 4 shows the value of peak flow Q (curve falling from the left to the right) and the ratio "axial stress at peak flow at x = 0" over "normal axial stress" (curve rising from the left to the right) for stenoses ranging from 1% to 99% diameter reduction. Flow Q decreases continuously from DS = 0% to DS = 100%, where it reaches (of course) zero. This means that the mean pressure in the arterial and venous nets upstream of the stenosis increases with stenosis severity. Furthermore, due to the progressive blood congestion inside the affected lung lobe, the peak pressures in the arteriolar, alveolar, and venous nets increase abnormally, too, so that these vessels are likely to get more and more damaged.

The second curve shows that the stress increase begins at roughly 30% diameter reduction and reaches twice the value of "normal" stress at high DS values. At these DS values, peak axial wall stress is thus three times greater than normal axial stress. For a vessel that normally does not have to bear such loads, this is certainly enormous and must therefore have deleterious consequences.

The curves depicted in Fig. 3 and 4 depend, of course, somewhat on the chosen parameter values. Nevertheless, since the values used in this numerical example are representative for PVs, the results are also representative of the magnitude of axial stress increases that can be generated by a stenosis located on an extraparenchymal PV.

## Discussion

The concepts and examples presented in this article show that already a moderate stenosis (diameter reduction of roughly 40%) located on the extraparenchymal part of a PV can generate an appreciable increase in axial wall stress in the proximal adjacent segment during peak forward flow. It must be pointed out that this result has been obtained using quite realistic data found in several publications. Since extraparenchymal veins are not firmly connected to the surrounding tissues, the axial force F that generates the cyclic stress increase cannot be absorbed by these tissues and is therefore present not only at the stenosis entrance but also in the whole extraparenchymal vein segment upstream of the stenosis. This segment is thus submitted to the same cyclic tractions.

In the calculated example, the driving pressure (P_PA _- P_LA_) was chosen equal to the value it would have in absence of a stenosis. Therefore, one can state that axial stress in the proximal segment begins to rise long before peak circumferential stress starts increasing. The fact that media and intima thickening is regularly found in intraparenchymal veins in case of severe PV stenosis [35] suggests that, ultimately, also mean circumferential stress becomes excessive in all veins of the considered lung lobe.

The supplementary axial wall stress generated by the stenosis appears to be comparable to, or even much greater than, "normal" axial stress. It is therefore quite likely to have deleterious effects on the vessel wall. Abnormal biological processes induced by excessive wall stresses are, indeed, increasingly reported in the literature [35-37], although mainly for arteries and the circumferential direction. With regard to increases in axial stress, one can imagine that these increases result for instance in circumferential endothelium tears, elastic membrane disruptions, smooth muscle cell hyperplasia, matrix changes, myofibroblast proliferation, etc.

The fact that venous pressures are lower than arterial pressures does not allow, of course, to conclude that increases in circumferential stress induced in extraparenchymal PV by pressure rises can never be excessive. But in patients treated by RF ablation, mean and peak pulmonary venous pressures are not abnormally high at the end of the procedure, and also not in the following days or weeks. Thus, circumferential stress should not be excessive during this period. One might, however, also argue that, before the intervention, the pressure in the proximal vein segment was very low over the whole cardiac cycle, due to the presence of the AF, and that the wall of the PV was, consequently, abnormally thin. The strong increase of systolic pressure in the proximal segment after restoration of the normal atrial function might thus immediately damage the PV via excessive circumferential stress. But, even if circumferential stress would strongly increase, this would not explain why stenoses appear usually in the extraparenchymal segment of the PV and not in intraparenchymal veins. It is thus unlikely that the progression of stenoses observed in patients is due to excessive circumferential stress. In contrast thereto, the concept of excessive axial wall stress proposed in this contribution provides a potential explanation for these well established facts, and it explains moreover why "non significant" stenoses (e.g. < 50% diameter reduction) are less prone to grow than severe ones, as was observed by several authors [14,18].

If the proposed explanation of the evolution of stenoses created by RF ablation should prove to be correct, then the best way to avoid the potential consequences of stenoses would be, of course, to find a method which does not create stenoses, or only small ones. This may perhaps be the case to a certain extent if lower ablation temperature can be used, or for particular techniques like cryoablation and ultrasound ablation [7,15,22]. Whether these techniques produce PV stenoses or not is not yet clear [38,40].

If better methods cannot be found, angioplasty without or with stent implantation will probably be unavoidable. For instance, Saad et al. already recommend PV stenosis dilatation for patients with luminal narrowing of more than 70%, irrespective of the presence or absence of symptoms [13]. The rationale of this recommendation resides in the unknown likelihood of developing pulmonary hypertension, as well as in the risk of lesion progression to total occlusion that could preclude the intervention [8,11,12,14]. However, in the late 5 years many authors have reported more or less negative results for angioplasty with or without stenting after RF catheter ablation or surgical repair of a pulmonary anomaly [10,19,41,42]. As far as the successful cases are concerned, it was pointed out that care was taken in the surgical or stenting procedure to achieve a minimal residual stenosis [42,43]. This is in full agreement with our results, which predict essentially that small or moderate stenoses are less prone to worsen than more severe ones.

If conventional stenting should remain unsatisfactorily because of an elevated rate of in-stent restenosis [44,45], the next step will probably be to try drug-eluting stents. It is still unknown whether they can prevent in-stent restenosis as well as they do this in coronary arteries. But the recommendation of achieving the smallest possible residual stenosis holds, of course, also for these stents because any residual lumen obstruction acts as a stenosis with regard to axial wall stress in the upstream vein segment. This recommendation is straight forwardly supported by recent results of Berkowitsch et al. who found that the relative reduction in PV diameter one day after the RF ablation was the strongest predictor of development of a severe PV stenosis [46]. Noteworthy is, furthermore, that our model is also in agreement with the observation that in patients presenting a stenosis approaching 70% of the lumen (= 90% reduction of the diameter), the flow to the affected lung is severely decreased [13]. As can be seen in Fig. 4, flow appears to have decreased by roughly 50% at DS = 70%, and by more than 90% at DS = 90%.

In the purely surgical domain, it is now well acknowledged that the sutureless marsupialization technique for the treatment of stenosed PV yields better result than the conventional technique [47-49]. According to our concept, this is probably due to the fact that the sutureless technique produces no post-operative stenosis, or a smaller one than a conventional anastomosis.

With regard to the vein segment (if any) situated downstream of the stenosis, it seems conceivable that the same mechanical phenomenon occurs in inverted direction during the retrograde flow phase (which is restored by the suppression of the AF). In this case, an important stenosis would actually have a deleterious effect not only on the upstream wall tissues but also on the downstream ones.

A further step would be now to test in vivo (for instance in dogs) the "predictions" derived from the theoretical model about the temporal evolution of PV stenoses in function of the "initial" DS. This could be done for instance by creating stenoses with a cuff placed around the PV.

## Conclusion

The concept of excessive axial stress proposed in this paper as an explanation for the negative time evolution of PV stenosis appears to be consistent with many well known facts about PV stenoses. It predicts that the higher the degree of stenosis after ablation, the greater the risk of an unfavorable evolution will be. This is in full agreement with the fact that severe PV stenoses seldom regress and even tend to evolve toward complete occlusion. With regard to angioplasty with or without stenting, it means that achieving the smallest possible residual stenosis is probably very important. Ideal would be, of course, to have an ablation technique that does not create PV stenoses.

## Authors' contributions

PAD and PAD adapted the general mathematical/physical model to the particular case of pulmonray vein stenosis. DCS provided the medical context of the article. All authors read and approved the final manuscript.

## Appendix

### A) Calculation of the pressure drop across the stenosis

According to Back et al. [24] the pressure drop ΔP across a stenosis can be expressed as

ΔP = ΔP_ef _+ ΔP_eM _+ ΔP_mf_

where ΔP_ef _and ΔP_eM _are pressure losses due to viscous and inertial forces, respectively, in the constriction cone, and ΔP_mf _the pressure loss due to viscous forces in the throat (Poiseuille law).

The formula for ΔP_ef _is:

(1) $$\Delta {\text{P}}_{\text{ef}}=\frac{128\;\eta {\text{ L}}_{\text{c}}{\text{ I}}_{\text{s}}}{\pi {\text{D}}_{\text{i}}^{4}}\text{Q}$$

where:

(2) $${\text{I}}_{\text{s}}=\frac{{\left({\text{D}}_{\text{i}}/{\text{D}}_{\text{s}}\right)}^{3}-1}{3(1-{\text{D}}_{\text{s}}/{\text{D}}_{\text{i}})}$$

The factor η is the viscosity of blood (3.5 mPa s), L_c _the length of the constriction cone (see Fig. 2), D_i _the inner diameter of the vein at the cross section x = 0, D_s _the inner diameter in the throat of the stenosis, and Q the flow.

The formula for ΔP_eM _is:

(3) ΔP_eM _= 0.5 β ρ[(1/A_s_)^2 ^- (1/A_i_)^2^] Q^2^,

where β is a factor we set equal to 1 (as proposed by Back et al. [24]). ρ is the density of blood, and A_i _and A_s _are the luminal areas at the stenosis entrance and in the throat, respectively.

The formula for ΔP_mf _is:

(4) $$\Delta {\text{P}}_{\text{mf}}=\frac{128\;\eta {\text{ L}}_{\text{m}}}{\pi {\text{D}}_{\text{s}}^{4}}\text{Q},$$

where L_m _is the length of the throat (see Fig. 2).

Writing the coefficients of Q and Q^2 ^in ΔP_ef_, ΔP_eM_, and ΔP_mf _in form of functions f_1_, f_2_, and f_3 _yields:

(5) ΔP = f_1 _Q + f_2 _Q^2 ^+ f_3 _Q = (f_1 _+ f_3_) Q + f_2 _Q^2^

### B) Calculation of flow Q

We express the relationship between pressures and hemodynamical resistances in the considered lung lobe by:

(6) P_PA _- P_LA _= R Q + ΔP = R Q + ΔP_ef _+ ΔP_eM _+ ΔP_mf_

where P_PA _is the pressure in the pulmonary artery at the time of peak pulmonary flow through the considered PV (not to confound with peak pulmonary artery pressure), P_LA _the left atrial pressure at this same time, Q the peak venous flow through the lobe, and R the hemodynamical resistance opposed by the lobe to flow Q (without the contribution of the stenosis). The summand RQ is thus the pressure drop in the lobe without the contribution of the stenosis. We have thus:

(7) P_PA _- P_LA _= (R + f_1 _+ f_3_) Q + f_2 _Q^2^

or, equivalently:

(8) f_2 _Q^2^+ (R + f_1 _+ f_3_) Q + (P_LA _- P_PA_) = 0

This quadratic equation allows to determine the value of Q when the values of f_1_, f_2_, f_3_, R, P_LA_, and P_PA _are known. One can thus calculate the value of Q for different degrees of diameter stenosis (for instance DS = 1, 5, 10, 15, ..., 90, 95, and 99%). The values of the coefficients f_1_, f_2_, and f_3 _are obtained from equations 1 to 4. They depend on the blood density and viscosity, and on the morphology of the stenosis.

The value of R is determined as follows. We consider the situation preceding the creation of the stenosis (DS = 0%). Denoting the peak flow through the considered lobe by Q_o_, and the corresponding resistance of the lobe by R_o_, we have P_PA _- P_LA _= R_o_Q_o_, so that R_o _= (P_PA _- P_LA_)/Q_o_. For Q_o_, we can set for instance Q_o _= 23.6 ml/s (= 1.4 liter/min). The hemodynamical resistance R_o _for DS = 0% being thus determined, one can then assume for instance that it remains unchanged when a stenosis appears, or have it decrease in a freely chosable manner to simulate a lowering of the hemodynamical resistance by compensatory vasodilatation. Inversely, one can also have R_o _increase to simulate a pathologic, progressive narrowing of the affected pulmonary vessels by increasing stenosis severity.

### C) Calculation of the increase in axial wall stress

To calculate the increase in axial stress generated by the stenosis in the wall cross section x = 0 at peak flow, one has to calculate first the supplementary axial force F that is generated in this cross section. As shown in [27], this is done using equations 1 to 4. It leads to:

$$\begin{array}{l} \text{F}=\frac{\pi ({\text{P}}_{\text{LA}}+\Delta \text{P})({\text{D}}_{\text{i}}^{2}-{\text{D}}_{\text{s}}^{2})}{4}-\frac{32\;\eta {\text{ L}}_{\text{c}}(2/{\text{D}}_{\text{s}}+{\text{D}}_{\text{s}}^{2}/{\text{D}}_{\text{i}}^{3}-3/{\text{D}}_{\text{i}})\text{ Q}}{3({\text{D}}_{\text{i}}-{\text{D}}_{\text{s}})}\\ -\frac{2\;\beta \;\rho (1/{\text{D}}_{\text{s}}^{2}+{\text{D}}_{\text{s}}^{2}/{\text{D}}_{\text{i}}^{4}-2/{\text{D}}_{\text{i}}^{2}){\text{ Q}}^{2}}{\pi }+\frac{32\;\eta {\text{ L}}_{\text{m}}\text{ Q}}{{\text{D}}_{\text{s}}^{2}} \end{array}$$

Division of the force F by the cross sectional area of the wall at x = 0 yields the supplementary axial stress that adds at peak flow to the "normal" axial stress of the wall.
